# Which factors accompany maternal acceptance-rejection in children with specific learning disabilities?

**DOI:** 10.1186/s12888-024-05584-8

**Published:** 2024-02-13

**Authors:** Ganime Ayar, Sıddıka Songül Yalçın, Özge Tanıdır Artan, Ahmet Kahveci, Esra Çöp

**Affiliations:** 1 Department of Pediatrics, Ministry of Health, Ankara City Hospital, Bilkent, Ankara, Turkey; 2 Faculty of Medicine, Department of Pediatrics Unit of Social Pediatrics and Unit of Child Development, Hacettepe University, Sıhhiye, Ankara, Turkey; 3 Department of Child Psychiatry, Ministry of Health, Ankara City Hospital, Bilkent, Ankara, Turkey

**Keywords:** Specific learning disability, Maternal acceptance/rejection, Maternal control, Children

## Abstract

**Background:**

Children with specific learning disabilities (SLDs) and their parents experience many problems that may influence their interactions. The study aimed to evaluate the maternal acceptance/rejection status of children with SLDs and their associations with sociodemographic characteristics, and problem behaviors.

**Methods:**

The Parental Acceptance-Rejection/Control Questionnaire (PARQ/C) and Strengths and Difficulties Questionnaire (SDQ) were applied to the mothers with children aged 7–17 years with a diagnosis of SLD.

**Results:**

Among 266 children enrolled, the mean age was 10.2 years, and 61.7% were male, the mean score was 30.4 for warmth/affection, 25.8 for hostility/aggression, 22.9 for indifference/neglect, 16.3 for undifferentiated rejection, 95.4 for the total PARQ, and 40.8 for the control scales. Generalized linear models revealed that maternal depression, poor family income, parental smoking, and presence of dysgraphia, and poor total difficulties and prosocial scores of SDQ subscales were associated with the maternal acceptance-rejection. There was an interaction between the maternal control subscale and the school success of the child.

**Conclusion:**

Mothers of children with SLDs had high maternal rejection scores which were associated with unfavorable characteristics of child and family. Early detection and giving appropriate support of these cases could improve the mother’s relationship with her SLD child.

## Introduction

Specific learning disabilities (SLDs), described as when the intelligence of a child is normal or above normal, but their acquired knowledge is behind those of their peers according to the education they are getting. Difficulties in cognitive processes for reading, mathematics, and written expression are present, despite their normal mental development [1]. The prevalence of SLD in school children from different languages and cultures is 5–15% [2]. In an epidemiological study 2174 primary school children in 2013–2014 in Turkey, the probable prevalence rates were found to be 13.6% [3]. On the other hand, SLD is considered to be relatively frequent and not known enough [1]. In particular, it is important to know all aspects of such a common disorder, the difficulties experienced by parents and their reflection on children, and the risks that increase these difficulties.

Parents have an important place in the lives of their children, and their support is important during the process of learning. When the need for a positive reaction is not met by their parents, it negatively impacts the functioning of an individual, and children and adolescents can respond to this situation in different ways, including emotionally and behaviorally [4].

The acceptance or rejection of parents during the childhood period affects the emotional, behavioral, and social-cognitive development of a growing child [5–7]. The presence of parental love, warmth, and care (acceptance) establishes the baseline for positive social and emotional development; however, rejection (coldness, insensitiveness, hostility, indifference, neglect) is more determinative for the psychological adjustment of the child in comparison with acceptance [8]. High parental acceptance and low rejection are related to positive developmental outcomes in the childhood period [9]. Besides, the level of control also evaluates the level of compliance that a child has with the rules and instructions of the parents [10].

Previous studies showed that the children of rejecting mothers had lower self-esteem, poor social communication skills, inadequate emotional skills [11, 12], and they experienced behavioral issues, such as low academic success [13]. Thus, when the maternal rejection scores of children with learning difficulties are high, this may mean that they will face more problems in their academic and social lives. However, there are limited reports on the status of parental rejection in cases having some learning problems [5, 14–17]. One of the factor affecting the emotional, behavioural, social and cognitive development of children is their level of acceptance or rejection by their parents and parental control behaviours.

Disability conditions, such as SLDs, are intricate and interdependent. Environmental factors encompass the physical, social, and attitudinal aspects in which individuals live and navigate their lives. An individual’s functioning level is a constantly evolving interplay between their health conditions, personal characteristics, and the environment they are in. Shifting from a medical model to a more comprehensive biopsychosocial perspective of disability underscores the critical role of environmental factors, particularly in the context of children and youth. One pivotal consideration is how the nature and intricacy of children’s surroundings undergo significant transformations during the various developmental stages of infancy, early childhood, middle childhood, and adolescence. Thus, it is imperative to give due attention to environmental factors as they have a pervasive influence on all aspects of a child’s life and may necessitate adjustments or modifications to ensure their well-being and development [18].

In this study, our primary objective is to assess maternal acceptance and rejection of children with SLDs and to investigate the potential correlation with various sociodemographic characteristics including the mother’s age, education level, financial situation, family characteristics, as well as the circumstances of the children including breastfeeding history, associated disabilities, behavioural problems, and siblings’ health status.

It is recognized that children diagnosed with Specific Learning Disabilities (SLD) often experience higher levels of maternal rejection. While many studies have delved into the relationship between parental acceptance-rejection behaviors and individual and family characteristics in families with children with SLD, they often focus on a limited set of factors at a time [5, 14–16]. In real-life situations, these factors frequently interact simultaneously, leading to mutual influences on their effects [17, 19]. Our hypothesis suggests that interactions within the ‘sick child-family-environment,’ encompassing factors such as parental education, socioeconomic status, family attributes, and issues related to children’s problem behaviors, will play a significant role in shaping maternal rejection.

Identifying the factors linked to parental non-acceptance behaviors can make a valuable contribution to the field of child development and support. By understanding these factors, anticipatory guidance programs can be tailored and designed to enhance the parent-child relationship, which is crucial for the well-being and development of children with SLDs. This research may provide insights into interventions and strategies that can help parents better understand and support their children with SLDs, ultimately fostering a more nurturing and constructive environment for their growth and development.

## Materials and methods

### Setting, participants and variables

This cross-sectional study comprised the participants volunteering to participate, having a diagnosis of SLDs, being between 7 and 17 years of age, and applying to the “Report Regulation Unit of Children Having Special Needs” between May 2019 and August 2019. All the patients having SLD were received follow-up and treatment by a team consisting of a child psychiatrist and a pediatrician at the Unit. Having associated chronic diseases, congenital abnormalities, comorbid psychiatric diagnosis, or being refugees were exclusion criteria. SLD diagnosis was made through a combination of psychiatric interviews evaluating reading, writing, and mathematics skills of the children, Teachers and Parents Information Form and the Specific Learning Difficulty Battery by a child and adolescent psychiatrists [20]. Children having IQ scores below 70 on Wechsler Intelligence Scale for Children-revised form and comorbid psychiatric diagnosis were excluded from the study.

After explaining the aim of the study to mothers of the children with SLDs, all the mothers that accepted to participate signed the informed consent form, and filled in a structured questionnaire that included questions about the mother, the child, and the family structure, and a Parental Acceptance-Rejection/Control Questionnaire (PARQ/C). For the illiterate mothers, both the constructed questionnaire and the PARQ/C were read aloud by the researcher, and the forms were filled in during a face-to-face interview.

Characteristics of the child (age, gender, gestational week, birth weight, birth type, breastfeeding, presence of paternal or maternal smoking, presence of hospitalization), maternal age at the birth of the enrolled child, maternal education level, family income, type of family (extended, single-parent, nuclear), number of children that the parents have, and presence of a sibling who has SLDs were acquired through the structured questionnaire. In order to facilitate understanding, family income was expressed in international dollars ($) based on the exchange rate at the time of writing this manuscript. In addition, the question “How do you think school success is? by adding to the survey, the school success evaluated by the mother was taken into account. The hospitalization history and diseases of the children were documented from the hospital records.

### Parental Acceptance-rejection questionnaire (parent form)

The Parental Acceptance-Rejection Questionnaire (PARQ) was developed by Rohner et al. (1978) to measure the perception of the parents about their rejection-acceptance of their children [21]. It was translated into Turkish and adapted by Erdem in 1990 [22]. The PARQ consisted of 60 items and 4 subscales: warmth/affection (W/A), hostility/aggression (H/A), indifference/neglect (I/N), undifferentiated rejection (UR). Then the control scale was added as the fifth scale. The recent form, called the Parental Acceptance-Rejection/Control Questionnaire (PARQ/C), consists of 73 items [10]. All the items in the questionnaire had 4 choices, comprising 4 = almost always, 3 = sometimes, 2 = rarely, and 1 = almost never. All of the items in the W/A scale were scored reversely and the sum of the scores taken from the four subscales yielded the total rejection score. High scores indicated high levels of rejection. The control subscale evaluates the behavior of the parents toward controlling and managing the behaviors of their children. In the current study, Cronbach’s Alpha value were 0.88 for WA, 0.83 for H/A, 0.80 for I/N, 0.75 for UR, 0.93 for total rejection scores, and 0.58 for control scales,.

#### Strengths and difficulties questionnaire (parent form)

The presence of problem behaviors in children was assessed using the Strengths and Difficulties Questionnaire (SDQ), Parent Version developed by Robert Goodman in 1997 [23]. It was translated into Turkish and validated by Güvenir et al. [24]. Strengths and Difficulties Questionnaire (SDQ) was performed for children with SLDs in a previous study [19]. The SDQ contains 25 items (5 referring to social skills and 20 to difficulties), categorized into 5 scales of 5 items each: emotional symptoms, conduct problems, hyperactivity/inattention, peer problems and pro-social behavior. A total difficulty score can be calculated by summing scores of four difficulties subscales, i.e., except for pro-social behavior. The sum of conduct problems and emotional symptoms describes the “internalizing problems”, while the sum of hyperactivity/inattention and peer problems describes the “externalizing problems” subgroup. The 90th percentiles (high limits) of a UK community sample [25] were selected as cut-off points for all subsections except for pro-social behavior and the bottom tenth percentile (low limit) as the cut-off point for pro-social behavior as given in the previous study [19].

#### Study sample size

The sample size was calculated as 256, using the single mean formula with standard deviation = 14.7 [14] at α = 0.05 and d = 1.8. The final sample size was determined to be 320 by estimating 25% of cases with inappropriate data.

### Statistical analysis

The distribution of parameters was examined using skewness, kurtosis, the Kolmogorov-Smirnov test, histograms. It was observed that the W/A, H/A, I/N, and UR sub-scores and the total PARQ score showed right-skewed distribution. The control scores showed a normal distribution. Descriptive statistics were given as the mean, standard deviation (SD), and geometric mean, while the categorical data were given in the form of numbers and percentages. The chi-square test was used to analyze the differences in frequencies of control types according to children’s characteristics.

Generalized linear models (GLMs, model: gamma log link) were used to determine interactions between the PARQ scores and each baseline characteristic of children and family with SLDs and to determine the relationship between mothers’ SDQ scores and mothers’ PARQ scores. The estimated marginal means (95% confidence intervals; CI) were calculated.

The PARQ/C subscores and total scores were evaluated with the GLM for the associations with maternal age at birth of index child, maternal education level, maternal depression, family income, family type, parental smoking, the age and gender of the child, birth weight and week, breastfeeding duration, and school success of the child, brother/sister status (singleton, brother/sister with SLDs, healthy brother/sister), dysgraphia and SDQ scores of child. *P* < 0.05 was accepted as statistically significant. IBM SPSS Statistics 22.0 was used for the analyses.

## Results

During the study period, 320 participants were admitted, 54 were excluded due to missing records and incomplete questionnaires. A total of 266 mother-child pairs (83.1%) were evaluated.

The mean age of mothers and children were 36.0 (± 5.5) years and 10.2 (± 2.2) years. The median age of children at the diagnosis of SLD was 7.5 years and 61.7% were male (Table 1).

**Table 1:** Characteristics of children with specific learning disability, *n* = 266

Characteristics		
Age, yr	mean ± SD	10.2 ± 2.2
Gender, male	n (%)	164 (61.7)
Children’s age at diagnosis of specific learning disability, yr	mean ± SD (median)	8.1 ± 1.7 (7.5)
Maternal age at birth of index age, yr	mean ± SD	25.8 ± 5.2
Paternal age at birth of index age, yr	mean ± SD	29.7 ± 5.7
Associated disabilities		
Dyslexia	n (%)	186 (69.9)
Dysgraphia	n (%)	202 (75.9)
Dyscalculia	n (%)	243 (91.4)
**Wechsler intelligence scale for children-revised form**		
Verbal scores	mean ± SD	75.4 ± 13.6
Performance scores	mean ± SD	88.8 ± 16.3
Full-scale scores	mean ± SD	82.1 ± 12.0
**Pathological scores of strengths and difficulties questionnaire scores**		
Emotional symptoms ≥ 5	n (%)	96 (36.1)
Conduct problems ≥ 4	n (%)	77 (28.9)
Hyperactivity-ınattention ≥ 8	n (%)	75 (28.2)
Peer problems ≥ 4	n (%)	157 (59.0)
Externalizing ≥ 12	n (%)	56 (21.1)
Internalizing ≥ 9	n (%)	99 (37.2)
Prosocial behavior ≤ 6	n (%)	73 (27.4)
Total difficulties ≥ 17	n (%)	128 (48.1)

SD: Standard deviation

For the children with SLDs, the mean (± SD) score was 30.4 (± 9.0) for W/A, 25.8 (± 7.6) for H/A, 22.9 (± 6.9) for I/N, 16.3 (± 5.2) for UR, 95.4 (± 23.8) for the total PARQ (t-PARQ), and 40.8 (± 4.8) for the control (Table 2). The W/A, H/A, I/N, t-PARQ and control scores of children under 10 and over 10 years of age with SLD, were 30.4/30.4, 26.0/25.7, 22.7/23.1, 16.4/16.2, 95.5/95.4, and 41.0/40.7, respectively (Table 3) and were not statistically significant.

**Table 2:** Mothers’ parental acceptance-rejection questionnaire (PARQ) and control scores in children with specific learning disability, *n* = 266

PARQ/C scores	Geometric mean	Mean	SD	25p	50p	75p
Warmth/ Affection	29.3	30.4	9.0	24.0	28.0	33.0
Hostility/Agression	24.8	25.8	7.6	20.0	24.0	30.0
Indifference/ Neglect	22.0	22.9	6.9	18.0	21.0	26.0
Undifferentiated rejection	15.6	16.3	5.2	12.0	15.0	19.0
Total-PARQ	92.8	95.4	23.8	77.8	90.0	108.0
Control scores	40.5	40.8	4.8	38.0	41.0	44.0

**Table 3:** The mean (95% CI) values of Parental Acceptance-Rejection Questionnaire (PARQ) scores according to maternal, family and children’s characteristics*

		Total (n)	W/A	*p*	HA	*p*	I/N	*p*	UR	*p*	T-PARQ	*p*	Control	*p*
Children’s age				0.983		0.710		0.614		0.770		0.962		0.639
	< 10 yr	112	30.4 (28.9–32.0)		26.0 (24.7–27.4)		22.7 (21.6–23.9)		16.4 (15.5–17.3)		95.5 (91.4–99.8)		41.0 (40.1–41.9)	
	≥ 10 yr	154	30.4 (29.1–31.7)		25.7 (24.6–26.8)		23.1 (22.1–24.1)		16.2 (15.5–17.0)		95.4 (91.9–99.0)		40.7 (39.9–41.4)	
Children’s gender				0.918		0.825		0.522		0.991		0.831		0.111
	Male	164	30.4 (29.2–31.7)		25.7 (24.7–26.8)		22.7 (21.8–23.7)		16.3 (15.6–17.1)		95.2 (91.8–98.7)		40.4 (39.7–41.2)	
	Female	102	30.3 (28.8–31.9)		25.9 (24.6–27.4)		23.2 (22.0-24.5)		16.3 (15.4–17.3)		95.8 (91.5-100.3)		41.4 (40.5–42.3)	
Maternal age at the birth of index child				0.737		0.436		0.782		0.904		0.966		0.487
	< 21 yr	45	30.1 (27.9–32.6)		26.3 (24.3–28.5)		23.2 (21.4–25.2)		16.3 (15.0-17.8)		96.0 (89.6-102.8)		41.4 (40.0-42.8)	
	21–30 yr	168	30.2 (29.0-31.5)		26.0 (24.9–27.1)		22.7 (21.8–23.7)		16.2 (15.5–17.0)		95.2 (91.8–98.6)		40.8 (40.1–41.5)	
	> 30 yr	53	31.2 (29.0-33.5)		24.7 (23.0-26.6)		23.3 (21.6–25.1)		16.6 (15.3–17.9)		95.8 (89.9-102.1)		40.3 (39.0-41.5)	
Maternal education				0.339		0.696		0.076		0.723		0.283		0.230
	< 8 yr	146	30.9 (29.4–32.3)		26.0 (24.7–27.2)		23.6 (22.5–24.7)		16.4 (15.6–17.3)		96.8 (93.0-100.7)		41.1 (40.4–41.9)	
	≥ 8 yr	120	29.8 (28.2–31.4)		25.6 (24.3–27.0)		22.1 (20.9–23.3)		16.2 (15.3–17.1)		93.7 (89.5–98.0)		40.4 (39.641.3)	
Maternal depression				**0.013**		0.661		0.150		0.856		0.303		0.932
	No	246	30.7 (29.7–31.7)		25.8 (24.9–26.7)		23.1 (22.3–23.9)		16.3 (15.7–16.9)		95.8 (93.0-98.7)		40.8 (40.2–41.4)	
	Yes	20	26.6 (23.7–29.9)		26.5 (23.5–29.9)		21.1 (18.7–23.8)		16.5 (14.5–18.8)		90.7 (81.8-100.5)		40.9 (38.8–43.0)	
Family types				0.572		0.575		0.311		0.284		0.359		0.082
	Two parents	195	30.2 (28.9–31.5)		25.7 (24.6–26.7)		22.7 (21.7–23.6)		16.1 (15.4–16.8)		94.6 (91.3–98.0)		41.1 (40.5–41.8)	
	Extended family or single parent	71	30.9 (28.8–33.0)		26.2 (24.5–28.0)		23.6 (22.0-25.2)		16.9 (15.7–18.1)		97.6 (92.1-103.2)		40.0 (38.9–41.1)	
Family income				**0.004**		**0.017**		**< 0.001**		**0.001**		**< 0.001**		0.638
	< 200 $	51	33.5 (31.2–36.1)		28.1 (26.0-30.3)		26.0 (24.1–28.0)		18.5 (17.1–20.0)		106.1 (99.6–113.0)		40.5 (39.2–41.8)	
	≥ 200 $	215	29.7 (28.6–30.7)		25.3 (24.4–26.2)		22.2 (21.4–23.0)		15.8 (15.2–16.4)		92.9 (90.1–95.8)		40.9 (40.2–41.5)	
Birth type				0.424		0.462		0.888		0.714		0.626		0.984
	Vaginal	169	30.1 (28.9–31.3)		25.6 (24.5–26.7)		22.9 (21.9–23.9)		16.4 (15.7–17.1)		94.9 (91.6–98.3)		40.8 (40.1–41.5)	
	C/S	97	30.9 (29.3–32.6)		26.2 (24.8–27.7)		23.0 (21.8–24.3)		16.2 (15.2–17.2)		96.3 (91.9-100.9)		40.8 (39.9–41.8)	
Birth weight and week				0.444		0.931		0.547		0.683		0.600		0.969
	< 2500 gr and/or < 38 wk	60	29.6 (27.3–31.9)		25.9 (24.0-27.8)		22.5 (20.7–24.2)		16.1 (14.8–17.4)		94.0 (88.0-100.0)		40.8 (39.6–42.0)	
	Normal wk and weight	206	30.6 (29.4–31.9)		25.8 (24.8–26.8)		23.1 (22.2–24.0)		16.4 (15.7–17.1)		95.8 (92.6–99.1)		40.8 (40.2–41.5)	
Breastfeeding duration				0.761		0.150		0.118		0.210		0.285		0.949
	< 12 mo	99	30.2 (28.7–31.8)		26.6 (25.2–28.1)		23.7 (22.5–25.0)		16.8 (15.8–17.8)		97.4 (93.0-102.0)		40.8 (39.8–41.7)	
	≥ 12 mo	167	30.5 (29.3–31.8)		25.3 (24.3–26.4)		22.4 (21.5–23.4)		16.0 (15.3–16.8)		94.3 (91.0-97.7)		40.8 (40.1–41.6)	
Hospitalization history				0.984		0.965		0.611		0.884		0.864		0.635
	No	233	30.4 (29.4–31.5)		25.8 (24.9–26.8)		22.8 (22.0-23.7)		16.3 (15.7–16.9)		95.3 (92.5–98.3)		40.8 (40.1–41.4)	
	Yes	33	30.4 (27.8–33.3)		25.8 (23.4–28.3)		23.5 (21.3–25.8)		16.4 (14.8–18.2)		96.1 (88.7-104.1)		41.2 (39.6–42.8)	
School achievement				0.079		0.881		0.916		0.635		0.823		**0.007**
	Unsuccessful	116	29.7 (28.3–31.2)		26.0 (24.7–27.3)		22.9 (21.8–24.1)		16.6 (15.6–17.3)		95.3 (91.3–99.4)		41.8 (41.0-42.7)^a^	
	Middle	102	31.8 (30.2–33.5)		25.5 (24.2–26.9)		23.0 (21.8–24.3)		16.0 (15.1–16.9)		96.3 (92.0-100.9)		39.9 (39.0-40.8)^b^	
	Good	48	29.1 (27.0-31.3)		26.0 (24.0-28.1)		22.6 (20.9–24.4)		16.3 (15.0-17.8)		93.9 (87.9-100.4)		40.4 (39.0-41.7)^ab^	
Brother/sister with SLD				0.250		0.221		0.110		0.524		0.157		0.652
	Healthy brother/sister	194	30.3 (29.2–31.4)		25.5 (24.5–26.5)		22.6 (21.8–23.5)		16.1 (15.5–16.8)		94.5 (91.4–97.6)		40.7 (40.1–41.4)	
	Brother/sister having SLD	56	31.6 (29.4–33.8)		27.3 (25.4–29.3)		24.4 (22.7–26.2)		17.0 (15.7–18.4)		100.2 (94.3-106.5)		40.8 (38.6–42.1)	
	Singleton	16	28.0 (24.6–31.9)		24.8 (21.7–28.4)		21.2 (18.5–24.2)		16.1 (13.9–18.7)		90.1 (80.4–101.0)		41.9 (39.5–44.2)	
Parental smoking				0.526		**< 0.001**		**0.010**		**0.002**		**0.004**		0.850
	No	102	30.0 (28.5–31.6)		23.7 (22.5–25.0)		21.7 (20.6–22.9)		15.2 (14.3–16.1)		90.6 (86.6–94.7)		40.9 (40.0-41.8)	
	Yes	164	30.6 (29.4–31.9)		27.1 (26.0-28.3)		23.7 (22.7–24.7)		17.0 (16.3–17.8)		98.5 (95.0-102.0)		40.8 (40.0-41.5)	
Dyslexia				0.868		0.950		0.860		0.528		0.916		0.351
	Absence	80	30.5 (28.8–32.4)		25.9 (24.3–27.5)		22.8 (21.5–24.2)		16.0 (15.0-17.1)		95.2 (90.4-100.2)		40.4 (39.3–41.5)	
	Presence	186	30.3 (29.2–31.5)		25.8 (24.8–26.8)		23.0 (22.1–23.9)		16.4 (15.7–17.1)		95.5 (92.4–98.8)		41.0 (40.3–41.7	
Dysgraphia				0.291		**0.010**		**< 0.001**		**0.001**		**0.001**		0.213
	Absence	64	29.5 (27.6–31.5)		23.9 (22.4–25.6)		20.0 (18.7–21.4)		14.7 (13.7–15.8)		88.1 (83.2–93.2)		40.2 (39.0-41.4)	
	Presence	202	30.7 (29.6–31.8)		26.4 (25.4–27.4)		23.8 (23.0-24.7)		16.8 (16.2–17.5)		97.8 (94.7-100.9)		41.0 (40.3–41.7)	
Dyscalculia				0.734		0.265		0.646		0.271		0.563		0.713
	Absence	23	31.0 (27.8–34.5)		24.3 (21.7–27.2)		22.3 (20.0–25.0)		15.3 (13.6–17.3)		92.9 (84.4-102.3)		41.2 (39.2–43.2)	
	Presence	243	30.3 (29.3–31.4)		26.0 (25.1–26.9)		23.0 (22.2–23.8)		16.4 (15.8–17.0)		95.7 (92.9–98.5)		40.8 (40.2–41.4)	

*Generalized linear models

^ab^Different superscripts denote significant differences between values

W/A: Warmth/ Affection, H/A: Hostility/Agression, I/N: Indifference/ Neglect, UR: Undifferentiated Rejection; T-PARQ: Total PARQ, SC: Strict control, SLD: specific learning disability

The perception of acceptance-rejection of the mothers did not differ based on the age of the children (< 10 years and ≥10 years) or gender (Table 3). The education level of the mother, family type, type of birth, birth weight, and week, breastfeeding duration, age of the mother at the birth, hospitalization history of the children, the presence of another child with SLDs in the family, family type, and the child’s school success did not show any significant association with the perceived acceptance-rejection of the mother.

The mean PARQ scores were meaningfully higher in the absence of maternal depression than in the presence of maternal depression for W/A (30.7 vs. 26.6, respectively; *p* < 0.05, Table 3). When compared with those having an income ≥ 200 $ (more than the national minimum wage), all the rejection scores (HA, I/N, UR, T-PARQ) were higher for families having an income < 200 $ (below the national minimum wage). Except WA, all the PARQ scores were meaningfully higher in the presence of parental smoking than those with no smoking.

When the parental control subscale was evaluated in children with SLDs, the mean control scores changed according to the school success of the child; children with low academic achievements had higher mean scores for the control subscales than children having middle and good school success (*p* = 0.007, Table 3).

## Associations between PARQ and SDQ subscales

High total rejection scores were detected in mothers with high total difficulties scores (102.2 vs. 89.1; *p* < 0.001, Table 4). WA scores of mothers with high conduct problems, peer problems, internalizing, poor prosocial behavior and total difficulties scores were also high (*p* = 0.009, *p* = 0.001, *p* = 0.003, *p* = 0.005 and *p* = 0.006, respectively). For HA, scores for total difficulties and difficulty scores in all sub-areas were significantly high. For I/N, UR, and tPARQ, the rejection scores of those with high difficulty scores except Hyperactivity-Inattention, were significantly high. Additionally, we found that those with high prosocial behavior and peer problems scores had meaningfully lower control scores (*p* < 0.05).

**Table 4:** The mean scores of Parental Acceptance-Rejection Questionnaire (PARQ) and Control Questionnaire according to scores of Strengths and Difficulties Questionnaire (SDQ) in children with specific learning disability*, *n* = 266

SDQ scores	W/A	*p*	HA	*p*	I/N	*p*	UR	*p*	T-PARQ	*p*	Control	*p*
Emotional symptoms		0.075		**0.024**		**< 0.001**		**0.003**		**0.002**		0.668
≥ 5	31.6 (30.0-33.3)		27.1 (25.7–28.7)		24.8 (23.5–28.2)		17.5 (16.5–18.6)		101.0 (96.5-105.8)		40.7 (40.0-41.4)	
< 5	29.7 (28.6–30.9)		25.1 (24.0-26.1)		21.9 (21.0-22.8)		15.6 (15.0-16.3)		92.3 (89.1–95.5)		40.7 (40.0-41.9)	
Conduct problems		**0.009**		**< 0.001**		**< 0.001**		**< 0.001**		**< 0.001**		0.128
≥ 4	32.5 (30.6–34.5)		29.1 (27.4–30.9)		25.7 (24.2–27.3)		18.6 (17.5–19.8)		105.9 (100.7-111.4)		40.1 (39.1–41.2)	
< 4	29.6 (28.5–30.7)		24.5 (23.6–25.4)		21.8 (21.0-22.6)		15.4 (14.8–16.0)		91.2 (88.3–94.1)		41.1 (40.4–41.8)	
Hyperactivity-Inattention		0.802		**0.027**		0.309		0.119		0.210		0.359
≥ 8	30.2 (28.4–32.1)		27.4 (25.7–29.2)		23.6 (21.8–23.6)		17.1 (16.0-18.3)		98.2 (93.2-103.6)		41.2 (40.2–42.3)	
< 8	30.5 (29.3–31.7)		25.2 (24.2–26.2)		22.7 (21.8–23.6)		16.0 (15.4–16.7)		94.3 (91.3–97.5)		40.6 (40.0-41.3)	
Peer problems		**0.001**		**< 0.001**		**< 0.001**		**< 0.001**		**< 0.001**		**0.023**
≥ 4	31.7 (30.4–33.0)		27.2 (26.1–28.4)		24.1 (23.1–25.1)		17.3 (16.6–18.1)		100.3 (96.8–104.0)		40.3 (39.5–41.0)	
< 4	28.5 (27.1–29.9)		23.8 (22.6–25.1)		21.2 (20.2–22.3)		14.8 (14.1–15.7)		88.4 (84.7–92.2)		41.6 (40.7–42.5)	
Externalizing		0.152		**< 0.001**		**0.001**		**< 0.001**		**< 0.001**		0.540
≥ 12	31.8 (29.7–34.1)		29.6 (27.6–31.8)		25.5 (23.7–27.4)		18.9 (17.5–20.3)		105.8 (99.6-112.3)		40.5 (39.2–41.7)	
< 12	30.0 (29.0-31.1)		24.8 (23.9–25.7)		22.2 (21.4–23.1)		15.6 (15.0-16.2)		92.7 (89.9–95.6)		40.9 (40.3–41.6)	
Internalizing		**0.003**		**< 0.001**		**< 0.001**		**< 0.001**		**< 0.001**		0.333
≥ 9	32.4 (30.7–34.1)		27.9 (26.4–29.4)		24.8 (23.5–26.2)		17.9 (16.9–19.0)		103.0 (98.5-107.7)		40.4 (39.5–41.4)	
< 9	29.2 (28.1–30.4)		24.6 (23.6–25.6)		21.8 (20.9–22.7)		15.4 (14.7–16.0)		91.0 (87.9–94.2)		41.0 (40.3–41.8)	
Prosocial behavior		**0.005**		**0.003**		**0.001**		**0.002**		**< 0.001**		**0.039**
≤ 6	32.8 (30.8–34.8)		28.0 (26.3–29.8)		25.2 (23.7–26.8)		17.8 (16.7–19.1)		103.8 (98.5-109.5)		39.8 (38.7–40.9)	
> 6	29.5 (28.4–30.6)		25.0 (24.0-25.9)		22.1 (21.2–22.9)		15.7 (15.1–16.4)		92.2 (89.3–95.3)		41.2 (40.5–41.9)	
Total difficulties		**0.006**		**< 0.001**		**< 0.001**		**< 0.001**		**< 0.001**		0.538
≥ 17	31.8 30.4–33.3)		28.0 (26.8–29.3)		24.7 (23.6–25.9)		17.8 (16.9–18.7)		102.2 (98.3-106.3)		41.0 (40.2–41.8)	
<17	29.1 (27.8–30.4)		23.8 (22.7–24.8)		21.3 (20.3–22.2)		14.9 (14.3–15.7)		89.1 (85.8–92.5)		40.6 (39.8–41.5)	

*Generalized linear models, Estimated marginal mean (95% CI)

W/A: Warmth/ Affection, H/A: Hostility/Agression, I/N: Indifference/ Neglect, UR: Undifferentiated Rejection; T-PARQ: Total PARQ.

### Multivariate analysis for subscales of PARQ and the characteristics of childhood and family

When all related variables were included in GLM, presence of maternal depression compared to absence, family income more than 200 $ compared to low income, unsuccessful school success compared to middle one, both pathologic TD and PsB scores compared to normal scores were found to be associated with high scores of WA subscales. GLM showed an interaction between high scores of HA subscales and variables including presence of parental smoking, both pathologic TD and PsB scores among studied variables. High scores of both IN and UR subscales were related with dysgraphia and SDQ scores. Low family income, presence of dysgraphia and pathologic TD scores showed an association with high T-PARQ scores. Among the studied variables, there was an association between only school success (unsuccessful vs. middle one) and control subscales of PARQ (Table 5).

**Table 5:** Multivariate analysis for determinants of the PARQ scores in children with specific learning disability*, *n* = 266

	W/A	*p*	HA	*p*	I/N	*p*	UR	*p*	T-PARQ	*p*	Control	*p*
Children’s age		0.978		0.412		0.916		0.663		0.730		0.434
<10 years	29.3 (26.7–32.1)		27.4 (25.0-30.2)		22.6 (20.6–24.7)		17.1 (15.5–18.9)		96.6 (89.3-104.5)		41.1 (39.4–42.9)	
≥ 10 years	29.3 (26.8–32.0)		26.7 (24.4–29.2)		22.6 (20.8–24.7)		16.8 (15.3–18.5)		95.7 (88.7-103.1)		40.6 (39.0-42.3)	
Children’s gender		0.993		0.962		0.523		0.926		0.872		0.172
Male	29.3 (26.8–32.0)		27.1 (24.7–29.6)		22.4 (20.5–24.4)		17.0 (15.4–18.7)		95.9 (88.9-103.4)		40.5 (38.8–42.1)	
Female	29.3 (26.7–32.2)		27.0 (24.6–29.7)		22.8 (20.8–25.0)		17.0 (15.3–18.7)		96.3 (89.1-104.2)		41.3 (39.5–43.1)	
Maternal age at the birth of child		0.675		0.244		0.574		0.902		0.939		0.154
<21 years	28.6 (25.5–32.1)		28.1 (25.1–31.5)		23.0 (20.6–25.7)		17.1 (15.1–19.3)		96.9 (88.1-106.7)		41.9 (39.8–44.2)	
21–30 years	29.2 (26.7–31.9)		27.4 (25.0–30.0)		22.1 (20.3–24.1)		16.8 (15.3–18.5)		95.7 (88.8-103.1)		40.6 (39.0-42.3)	
>30 years	30.0 (27.2–33.2)		25.8 (23.3–28.4)		22.7 (20.6–25.0)		17.1 (15.3–19.0)		95.8 (88.1-104.2)		40.0 (38.2–41.9)	
Maternal education		0.811		0.512		0.186		0.789		0.440		0.152
<8 years	29.4 (26.7–32.4)		27.4 (24.8–30.2)		23.1 (21.0-25.4)		17.1 (15.4–19.0)		97.2 (89.6-105.5)		41.3 (39.5–43.2)	
≥8 years	29.2 (26.7–31.8)		26.8 (24.5–29.2)		22.1 (20.3–24.0)		17.0 (15.4–18.5)		95.1 (88.4-102.3)		40.4 (38.8–42.1)	
Maternal depression		**0.015**		0.392		0.187		0.854		0.359		0.506
No	31.5 (29.4–33.8)		26.4 (24.6–28.2)		23.5 (22.0-25.1)		16.9 (15.7–18.2)		98.4 (92.9-104.3)		40.5 (39.2–41.8)	
Yes	27.2 (23.9–31.0)		27.8 (24.4–31.7)		21.7 (19.1–24.7)		17.1 (14.8–19.7)		93.9 (84.1-104.8)		41.2 (38.8–43.8)	
**Family types**		0.687		0.702		0.831		0.674		0.889		0.152
Two parents	29.5 (26.8–32.5)		27.3 (24.7–30.1)		22.5 (20.5–24.7)		16.8 (15.2–18.7)		96.4 (88.8-104.6)		41.4 (39.6–43.3)	
Other	29.0 (26.5–31.8)		26.9 (24.5–29.4)		22.7 (20.8–24.8)		17.1 (15.5–18.9)		95.9 (88.8-103.6)		40.3 (38.7–42.1)	
Family income		**0.006**		0.096		**0.022**		**0.037**		**0.007**		0.767
< 200 $	31.2 (28.0-34.7)		28.1 (25.2–31.3)		23.8 (21.4–26.4)		17.9 (15.9–20.1)		101.3 (92.6-110.9)		40.7 (38.8–42.8)	
≥ 200 $	27.5 (25.3–29.9)		26.0 (23.9–28.4)		21.5 (19.7–23.3)		16.1 (14.7–17.7)		91.2 (84.9–98.0)		41.0 (39.4–42.6)	
**Birth weight and week**		0.691		0.349		0.819		0.920		0.949		0.844
<2500 gr and/or < 38 wk	29.1 (26.3–32.1)		27.6 (24.9–30.5)		22.5 (20.4–24.8)		17.0 (15.3–19.0)		96.2 (88.4-104.7)		40.8 (39.0-42.7)	
Normal wk and weight	29.5 (27.0-32.2)		26.6 (24.4–29.0)		22.7 (20.8–24.7)		16.9 (15.4–18.6)		96.0 (89.2-103.3)		40.9 (39.3–42.6)	
Breastfeeding duration		0.416		0.399		0.151		0.390		0.581		0.557
<12 months	28.9 (26.3–31.7)		27.4 (25.0-30.1)		23.1 (21.1–25.3)		17.2 (15.6–19.1)		96.9 (89.6-104.8)		41.0 (39.3–42.8)	
≥12 months	29.7 (27.1–32.5)		26.7 (24.4–29.2)		22.1 (20.2–24.1)		16.7 (15.2–18.4)		95.4 (88.4-102.9)		40.7 (39.0-42.4)	
School achievement		**0.047**		0.507		0.854		0.510		0.614		**0.014**
Unsuccessful	28.4 (25.9–31.0)^a^		26.5 (24.2–29.0)		22.3 (20.5–24.4)		17.0 (15.4–18.7)		94.4 (87.5-101.8)		42.0 (40.3–43.8)^a^	
Middle	30.9 (28.1–33.9)^b^		26.8 (24.4–29.4)		22.6 (20.6–24.7)		16.5 (14.9–18.3)		96.9 (89.6-104.9)		40.1 (38.4–41.9)^b^	
Good	28.7 (25.7–32.0)^ab^		27.9 (25.0-31.2)		22.9 (20.6–25.4)		17.5 (15.5–19.7)		97.1 (88.5-106.5)		40.5 (38.5–42.6)^ab^	
**Brother/sister with SLD**		0.736		0.827		0.801		0.924		0.884		0.538
Healthy brother/sister	29.7 (27.4–32.2)		26.8 (24.8–29.1)		22.6 (20.9–24.5)		16.8 (15.4–18.3)		96.2 (89.9-102.9)		40.3 (38.9–41.8)	
Brother/sister having SLD	29.9 (27.2–32.9)		27.5 (25.0-30.3)		23.1 (21.1–25.3)		16.8 (15.2–18.6)		97.5 (90.0-105.6)		40.5 (38.8–42.3)	
Singleton	28.2 (24.3–32.8)		26.8 (23.1–31.2		22.1 (19.1–25.5)		17.3 (14.7–20.3)		94.7 (83.5-107.5)		41.7 (39.0-44.7)	
**Parental smoking**		0.783		**0.008**		0.362		0.193		0.196		0.845
No	29.4 (26.7–32.4)		25.9 (23.5–28.5)		22.3 (20.3–24.4)		16.6 (15.0-18.4)		94.4 (87.1-102.3)		40.8 (39.0-42.6)	
Yes	29.2 (26.7–31.8)		28.3 (25.9–30.9)		22.9 (21.1–25.0)		17.4 (15.8–19.1)		97.9 (90.9-105.4)		40.9 (39.3–42.6)	
**Dysgraphia**		0.751		0.071		**< 0.001**		**0.013**		**0.020**		0.291
Absence	29.1 (26.3–32.2)		26.1 (23.6–28.9)		21.0 (19.1–23.2)		16.1 (14.5–18.0)		92.6 (85.0-100.8)		40.5 (38.7–42.4)	
Presence	29.5 (27.0-32.1)		28.0 (25.7–30.5)		24.3 (22.4–26.4)		17.8 (16.3–19.6)		99.8 (92.9-107.3)		41.2 (39.6–42.9)	
**SDQ scores**		**0.012**		**< 0.001**		**< 0.001**		**< 0.001**		**< 0.001**		0.105
TD < 17, PsB > 6	27.2 (24.9–29.7)^a^		24.5 (22.4–26.8)^a^		20.5 (18.8–22.4)^a^		15.5 (14.0–17.0)^a^		87.8 (81.4–94.7)^a^		41.4 (39.7–43.1)	
TD < 17, PsB ≤ 6	29.7 (26.2–33.6)^ab^		26.7 (23.5–30.3)^ab^		22.4 (19.9–25.3)^ab^		16.0 (14.0-18.3)^ab^		95.0 (85.5-105.5)^ab^		40.9 (38.6–43.3)	
TD ≥ 17, PsB > 6	29.1 (26.4–32.0)^ab^		28.0 (25.5–30.9)^b^		22.7 (20.7–25.0)^b^		17.5 (15.8–19.4)^bc^		97.4 (95.5-105.6)^b^		41.7 (39.9–43.5)	
TD ≥ 17, PsB ≤ 6	31.4 (28.0-35.1)^b^		29.3 (26.2–32.8)^b^		24.9 (22.3–27.8)^b^		19.2 (17.0-21.7)^c^		105.0 (95.5-115.5)^b^		39.6 (37.6–41.6)	

*Generalized linear models

W/A: Warmth/ Affection, H/A: Hostility/Agression, I/N: Indifference/ Neglect, UR: Undifferentiated Rejection; T-PARQ: Total PARQ.

TD: Total difficulties, PsB: Prosocial behavior

^abc^Different superscripts denote significant differences between values

## Discussion

In this study, perceived rejection levels of the mothers towards their children having SLDs and correlated factors were investigated. Previous studies determined that children with problems perceive rejection by the mother more than children without problems and their psychological adjustment is worse than others [14, 26]. This study has obtained higher scores compared to previous studies involving healthy preschool children [27]. However, it has also achieved lower scores when compared to adolescents with conduct disorders [26]. Compared to the current study, a study with 134 adolescents reported much higher mean PARQ scores of the mothers of adolescents with conduct disorders (54.4 for W/A, 32.4 for H/A, 31.6 for I/N, 22.8 for UR, and 132.3 in total) [26]. As seen, maternal total scores for adolescence without conduct disorders [26] were similar to the current study. In another study, when the mean PARQ scores of 76 healthy preschool children were examined, it was found that they were lower than the mean scores of our children with SLD [27].

A study also stated that when 27 children with SLDs and the control group without SLDs were compared, the perceived maternal rejection scores were higher in children with SLDs (92.8 vs. 82.8), which were similar to the results determined herein [15]. In another study of children with SLDs, it was observed that children with SLDs had a higher perceived rejection from their mothers and more psychological adjustment issues than children with diabetes [14].

There was no difference in the PARQ scores with regards to gender in children with SLDs. Similarly, a past study with a smaller sample size showed no significant difference in the perceived maternal rejection level based on gender [15]. We found that if you have a previous history of maternal depression and the monthly income was lower, the W/A scores of the mothers were higher. In the current study, the scores were higher for H/A, I/N, and U/R, in addition to W/A, in families with lower monthly incomes, and this showed that low socioeconomic status was associated with the perceived rejection of the mothers of children with SLDs, in general, as supported by the literature [28]. A study among 171 university students also showed that those who belong to high socioeconomic status perceived more parental acceptance than students belonging to low socioeconomic status [29]. Compared to schizophrenia patients from middle and high socioeconomic status families, those from low socioeconomic status families perceived their mothers and fathers as more cold, neglectful, and rejecting, in their childhood [28]. Similarly, the rejection scores were higher in mothers of poor families who had children with SLDs, and poverty disrupted the mother-child relationship.

In a study conducted on 12–16-year-old adolescents, it was determined that perception of little parental warmth was correlated with lower levels of school adjustment, and children from families with high neglect or control levels had the highest school maladjustment [30]. Again, in different studies, academic success and the psychological adjustment of the children had a significant relationship with parental acceptance [31, 32]. In the current study, the rejection scores of the mothers of children with SLD were not related to academic achievement. However, academic achievement affected mothers’ control scores. Similarly, children of mothers with high control scores had lower academic success. In a study conducted with 1285 adolescents between the ages of 12–16, Jauraguizar et al. showed that students from authoritative families presented the highest levels of school maladjustment. Recent studies on parenting styles have found that indulgent parenting styles characterized by warmth are more effective than the authoritative style characterized by strictness [30]. Additionally, in families with high parental control, constant governance and protection of the children, and parents deciding everything for the children are factors that hinder the development of autonomy in children. Overprotected children fail to develop self-esteem and social skills [33]. In our study, another reason for children with SLD and low school success to have higher maternal control scores could be the belief that children’s academic success and sense of responsibility will improve as the parents’ control over them increases.

It was also observed that parental smoking associated with high maternal rejection scores; hence, mothers who smoked had a problem accepting their children. This situation may cause the failure of proper emotional attachment with their children. There is evidence in the literature that women who smoke breastfeed for shorter periods and had difficulty bonding with their infants than women who do not smoke [34]. It would be expected that breastfeeding mothers experience decrease on the craving for smoking and overall tobacco use, considering the fact that mothers who breastfeed secrete oxytocin, and it was observed in the literature that the smoking desire and consumption decreased in smokers who were given intranasal oxytocin [35]. Moreover, although the evidence regarding the physiological mechanism is not sufficient, negative effects of smoking on oxytocin release are reported in the breastfeeding literature [36]. Experiencing mother-infant attachment problems may be indirectly related to oxytocin due to not breastfeeding and smoking. However, our limitation here is that in children with smoke exposure, maternal smoking and environmental smoke exposure were not evaluated separately. Negative effects of smoke exposure in pre and postnatal period is well-known, and parents are being informed and warned about this issue. We think that parents who do not protect their babies from smoke exposure despite the warnings have a high probability of having elevated rejection scores for their children from the beginning.

Previous studies provide evidence of an association between breastfeeding and mother-infant attachment. In a study conducted to determine the risk factors associated with maltreatment of children, has found higher risk of victimization in children who were breastfed for less than 6 months [37]. In the current study, there is not a meaningful difference regarding maternal rejection between the babies that were breastfed for more than 12 months and those who were breastfed for less. On the other hand, our limitation here is that we did not evaluate the maternal rejection scores related for six months and later and the total duration of breastfeeding.

In our study, the mother’s history of depression decreased the mother’s warmth scores even though it did not affect the total rejection scores. Rohner and Britner in 2002 stated that there is a correlation between parental acceptance-rejection and mental health problems such as depression [38]. A study by Kim et al. investigated the mediating role of parental acceptance-rejection in the relationship between parental depressive symptoms and children’s mental health. They found that, for mothers and fathers, parental rejection was a powerful mediator in the association between parenting depressive symptoms and child psychosocial problems [39]. Örün et al. found in their study that mother-infant bonding and later mother-child relationship are associated with maternal psychopathologies, especially maternal depression [40]. In this regard, our results were coherent with the literature [38–40].

In some studies, it was stated that the perceived maternal acceptance-rejection changed according to the culture, and the education of the mother [41]. In the current study, the maternal education levels and the rejection or permissibility-strictness levels in the control subscale had no relation. The rejection factor is highly determinant, in comparison with acceptance, for the appearance of psychological and behavioral problems in children.

When we compared the strength and difficulties of mothers with their acceptance-rejection scores, we observed that total rejection scores were meaningfully high in mothers with high total difficulties scores. When subgroups are taken into consideration, in all subgroups except hyperactivity, mothers with high difficulty scores also had high rejection scores. Also, as a very important detail, maternal rejection scores were higher if prosocial behaviors were less and total difficulties were high. On account to the fact that we could not find another study on this topic in the literature, we could not make any comparisons. We think that our study is important because it is the first study that identifies maternal rejection and the risk factors for this rejection, and also compares the strengths and difficulties of mothers with acceptance-rejection scores in children.

One limitation is that this study was solely dependent on maternal statements. Due to its design, this study could not establish cause-and-effect relationships. The data were collected at a single point in time. In addition, another limitation of the study is that the importance of maternal support network and father absence was not studied. However, the current study included a good sample size for with several baseline characteristics and problematic behaviour of children with SLD and studied the association with maternal acceptance and rejection for the first time. Further longitudinal studies can be planned with the outcome of this study.

## Conclusion

In conclusion, a low level of family income and parental smoking, caused an increase in maternal rejection scores. And the lack of academic success of the child with SLD was associated with the high strict control scores of the mothers. In addition, as the mothers’ strength and difficulties scores increased, the mothers’ rejection scores also increased. Our study offers valuable insights and guidance toward achieving Sustainable Development Goals [42], including eradicating poverty (Goal 1), promoting good health and well-being (including mental health) (Goal 3), and ensuring quality education (Goal 4) both in the present and for future planning for SLD. Keeping the accompanying factors of maternal rejection under control is necessary for obtaining good results in SLD monitoring. In the future, research should focus on not only special education in SLDs but also on developing a parenting program that includes ways to increase parental acceptance and decrease rejection. Decreasing parental rejection and increasing acceptance by teaching the ways for expressing love verbally and nonverbally or decreasing hostility by emotion management will increase success for solving this issue. In addition, policy makers and schools should pay more attention to supporting lower socioeconomic background parents to avoid the potential negative effects of parental rejection.

## Data Availability

The datasets used and/or analyzed during the current study are available from the corresponding author on reasonable request (siyalcin@hacettepe.edu.tr).
